# Coccidioidomycosis in Northern Arizona: an Investigation of the Host, Pathogen, and Environment Using a Disease Triangle Approach

**DOI:** 10.1128/msphere.00352-22

**Published:** 2022-08-16

**Authors:** Heather L. Mead, Daniel R. Kollath, Marcus de Melo Teixeira, Chandler C. Roe, Carmel Plude, Nivedita Nandurkar, Chelsea Donohoo, Brettania L. W. O'Connor, Joel Terriquez, Paul Keim, Bridget M. Barker

**Affiliations:** a Northern Arizona University, Flagstaff, Arizona, USA; b Translational Genomics Research Institute (TGen), Phoenix and Flagstaff, Arizona, USA; c University of Brasilia, Brasilia, Brazil; d Northern Arizona Healthcare, Flagstaff, Arizona, USA; e Coconino County Health Department, Flagstaff, Arizona, USA; University of Georgia

**Keywords:** coccidioides, Valley fever, disease triangle, Southwestern United States, endemic mycoses, Northern Arizona, phylogenetic analysis, environmental microbiology

## Abstract

*Coccidioides immitis* and *Coccidioides posadasii* are the etiological agents of coccidioidomycosis (Valley fever [VF]). Disease manifestation ranges from mild pneumonia to chronic or extrapulmonary infection. If diagnosis is delayed, the risk of severe disease increases. In this report, we investigated the intersection of pathogen, host, and environment for VF cases in Northern Arizona (NAZ), where the risk of acquiring the disease is much lower than in Southern Arizona. We investigated reported cases and assessed pathogen origin by comparing genomes of NAZ clinical isolates to isolates from other regions. Lastly, we surveyed regional soils for presence of *Coccidioides*. We found that cases of VF increased in NAZ in 2019, and *Coccidioides* NAZ isolates are assigned to Arizona populations using phylogenetic inference. Importantly, we detected *Coccidioides* DNA in NAZ soil. Given recent climate modeling of the disease that predicts that cases will continue to increase throughout the region, and the evidence presented in this report, we propose that disease awareness outreach to clinicians throughout the western United States is crucial for improving patient outcomes, and further environmental sampling across the western U.S. is warranted.

**IMPORTANCE** Our work is the first description of the Valley fever disease triangle in Northern Arizona, which addresses the host, the pathogen, and the environmental source in the region. Our data suggest that the prevalence of diagnosed cases rose in 2019 in this region, and some severe cases necessitate hospitalization. We present the first evidence of *Coccidioides* spp. in Northern Arizona soils, suggesting that the pathogen is maintained in the local environment. Until disease prevention is an achievable option via vaccination, we predict that incidence of Valley fever will rise in the area. Therefore, enhanced awareness of and surveillance for coccidioidomycosis is vital to community health in Northern Arizona.

## INTRODUCTION

The “disease triangle” is a concept that considers how the interaction among host, pathogen, and the environment work together to influence disease outcome. Infectious diseases emerge when a vulnerable host, virulent pathogen, and favorable environmental conditions complete the triangle (1). This framework has been applied to a variety of diseases, perhaps most famously to the Irish potato famine (2, 3). Similarly, during the global COVID-19 pandemic, the idea has been applied to determine the reservoir of SARS-CoV-2 (4–6). The disease triangle concept is a useful tool to predict and mitigate future impacts to human health. One environmentally-acquired fungal infection of concern is coccidioidomycosis (Valley fever, VF), caused by two *Coccidioides* species. Cases of this disease have been increasing nationwide for decades (7–9).

VF in humans can be self-limited, requiring little to no medical care, or chronic, causing years of treatment and/or lifelong symptoms (10). Infection occurs when aerosolized environmental arthroconidia are inhaled by a susceptible host, and VF typically manifests as a respiratory infection (11). In severe cases, dissemination to extrapulmonary sites occurs and may require lifelong antifungal therapy (10, 11). Determining annual disease burden is challenging, because many states do not track or report cases and asymptomatic infections are unlikely to be reported, but estimates range from 150,000–350,000 cases/year in the United States (8, 9). Therefore, reported cases underestimate actual cases due to individuals not seeking medical care, misdiagnosis, and underreporting (8, 12–14). Preventing infection is complicated because the amount of detectable fungal spores in the air likely fluctuates daily; therefore, public health warnings based on environmental detection via air sensors could be inaccurate and/or delayed (15, 16). The factors that contribute to host susceptibility, such as genetics and immunological status, are not completely resolved; thus, identification of all susceptible individuals is not possible currently (17, 18). Finally, despite decades of research, a vaccine does not exist (19, 20). Currently, the best tool to combat VF is improved awareness for patients and health care professionals, so that correct treatment can be initiated (21–23).

Retrospective studies have identified host-specific factors that contribute to disease outcome (18, 24–28). This information is incomplete because patients with asymptomatic or acute cases may not seek medical care and thus are likely not included in analysis of risk. Evidence suggests individuals of African or Filipino descent are a risk group for severe disease, in particular disseminated infection (28). Recently, it has been identified that Native American populations are at increased risk of dissemination and hospitalization rates (14). The underlying mechanisms responsible for increased disease vulnerability are undefined, and observations are complicated but do not nullify the indication of increased risk, but rather validate the need for in-depth studies that consider these aspects (29, 30). To further complicate a complete understanding of host susceptibility, severe VF can occur in otherwise healthy individuals with no apparent immunological impairment (31–33). In general, host-specific aspects that dictate VF disease outcomes are undefined or incomplete, which prevents identification of high-risk populations (9, 18).

The evolution of a host-specific life cycle, which is distinct from the environmental life cycle, contributes to *Coccidioides’* virulence potential (34). When the environmental arthroconidia are inhaled, they develop into spherules, which are uniquely adapted for survival and proliferation in the host (35–38). The *Coccidioides* genus is comprised of two species, *C. immitis* and *C. posadasii* (39, 40), each with defined subpopulations and biogeographic patterns (41–43). Currently, the scale of phenotypic variation among isolates is unknown, although differential virulence has been shown (31, 44–47). There is a significant lack of knowledge of the basic biology of the pathogen, genes associated with pathogenesis, and environmental parameters that support or restrict growth and formation of infectious propagules (17, 48–51).

The environmental niche that supports the growth and proliferation of *Coccidioides* is undefined. A mammalian endozoan model has been proposed, which suggests that fungi survive within desert mammals asymptomatically and return to the saprobic form upon host death (52, 53). The organism has been detected in wild mammals, burrows, and archeological sites that are likely enriched with animal-derived organic matter (40, 54–57). The endemic region includes arid to semi-arid areas of the Americas from the southwestern United States to Argentina (41, 58). Recently, VF cases were locally acquired in the eastern arid region of Washington State, with confirmed environmental detection (59, 60). Climate models of the environmental distribution of *Coccidioides* predict the expansion of the organism across most of the western United States in response to climate warming (61, 62). Regions that are not traditionally considered endemic could serve as suitable habitat for *Coccidioides*, and cases of VF cases are predicted to rise (63).

Historically, Arizona has reported the highest number of diagnosed cases of VF each year, except in 2018 when surpassed by California (7, 9). In moderate- to low-incidence regions of disease such as Northern Arizona (NAZ), reported cases of VF are lower but still prevalent (64). Infection is often attributed to travel or prior residence in highly endemic regions. Like Southern Arizona, case incidence in NAZ has been on the rise over the last 2 decades (64, 65). However, the environment and host demographics in NAZ are different from southern counties. The region is predicted to support the pathogen’s growth in the near future, but little focus has been placed on the current disease burden of the region (62).

Herein, we conduct the first focused investigation of VF in humans in NAZ, which serves as a template to investigate the organism and disease in other suspected VF endemic areas. We identify susceptible populations, determine pathogen genomics, and discover possible local origins of infection. Our goal was to document the region-specific implications for human health regarding VF, to propose a blueprint for future investigations, and to point out the need for public health awareness campaigns beyond the current established areas of high incidence.

## RESULTS

### Diagnosed Valley fever cases in Northern Arizona counties.

**FIG 1:**
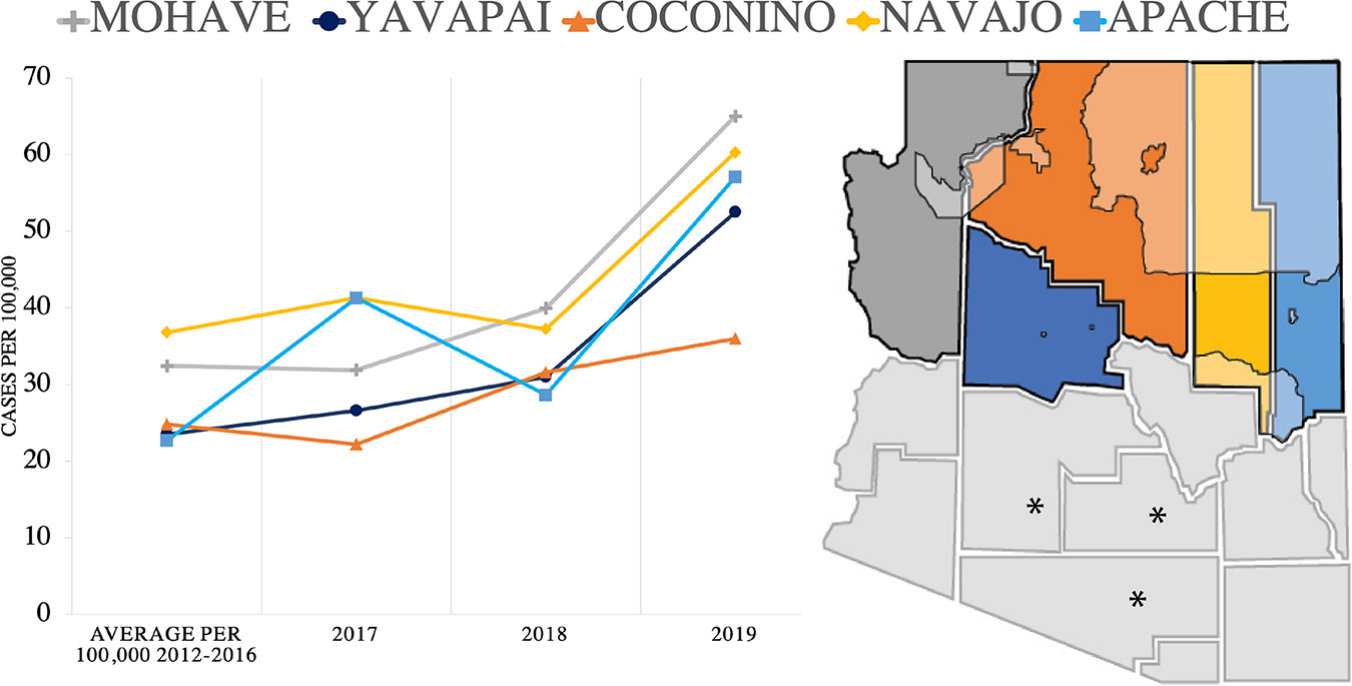
Northern Arizona county location and Valley fever cases. Yearly, the number of cases per 100,000 population increased compared to previous averages in all Northern Arizona counties. Geographic location of northern Arizona counties. Left to right: Mohave, Yavapai, Coconino (largest), Navajo, and Apache. Tribal land located in Northern Arizona counties are shaded. The southern counties that experience the highest yearly case counts per 100,000 are noted with asterisks.

NAZ counties are experiencing increased cases of VF per 100,000 population compared to the previous trends defined by the Health Department (Fig. 1). We utilized 2017–2019 data as this time frame coincides with the hospital data available. In Mohave, Yavapai, and Apache counties, the number of cases per 100,000 population doubled by 2019 in comparison to previous years’ averages (Fig. 1). In general, all counties in Arizona are reporting an increase in diagnosed cases each year and an increase in incidence per 100,000 population (Tables S3 and S4 in the supplemental material) with a few exceptions. The age and sex of individuals testing positive for VF in northern counties are similar to statewide observations (Table S5).

10.1128/msphere.00352-22.3 TABLE S3 Reported cases/100,000 population Download Table S3, DOCX file, 0.02 MB. Copyright © 2022 Mead et al. 2022 Mead et al. https://creativecommons.org/licenses/by/4.0/ This content is distributed under the terms of the Creative Commons Attribution 4.0 International license.

10.1128/msphere.00352-22.4 TABLE S4 Reported cases in Arizona. Download Table S4, DOCX file, 0.02 MB. Copyright © 2022 Mead et al. 2022 Mead et al. https://creativecommons.org/licenses/by/4.0/ This content is distributed under the terms of the Creative Commons Attribution 4.0 International license.

10.1128/msphere.00352-22.5 TABLE S5 Observed incidence of gender and age for Valley fever patients in northern Arizona counties. Download Table S5, DOCX file, 0.02 MB. Copyright © 2022 Mead et al. 2022 Mead et al. https://creativecommons.org/licenses/by/4.0/ This content is distributed under the terms of the Creative Commons Attribution 4.0 International license.

### Valley fever hospitalizations in Coconino County.

**FIG 2:**
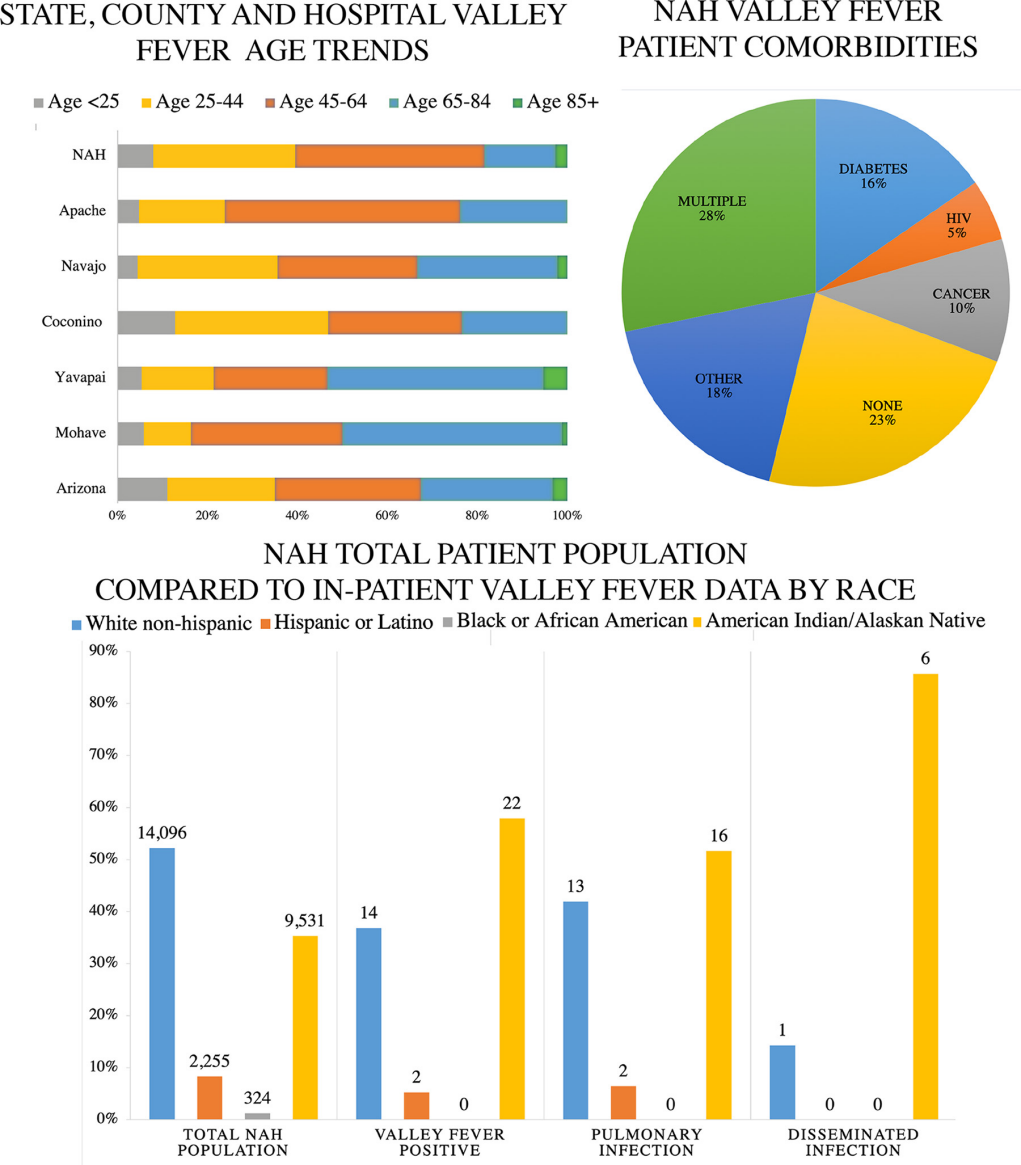
Northern Arizona Valley fever data demographics. (A) Age of patients diagnosed with Valley fever in Arizona during 2018, Northern Arizona counties during 2018, and regional hospital (NAH) between 13 July 2017 and 31 December 2018. (B) Among hospitalized Valley fever patients at NAH, 23% of patients had no documented pre-existing conditions. The other 77% had existing comorbidities such as diabetes (16%), HIV (9%), cancer (10%), and several multiple immunocompromising conditions. (C) The total population treated at NAH is displayed next to inpatient Valley fever cases for reference. Out of the 38 patients diagnosed with Valley fever from 13 July 2017 to 31 December2018, pulmonary infection occurred in 31 (82%) cases, and dissemination to extrapulmonary locations occurred in 7 (12%) of the cases. There were 14 white (36.8%), 2 Hispanic (5.2%), and 22 American Indian/Alaskan Native (57.8%) individuals.

We surveyed all inpatient records at the regional hospital (Northern Arizona Healthcare; NAH) for an 18-month period (13 July 2017 to 31 December2018). This regional medical center is in Coconino County but admits patients from other NAZ counties. During the time frame we investigated, there were 124 in-patients screened for VF, and 38 of them were positive for VF (+IgM N = 28, +CF titer N = 16). Because statewide VF hospitalization demographic data is not publicly available, we compared these inpatient cases to all reported VF case in Arizona with respect to sex and age (Table S5, Fig. 2A). In Arizona, reported cases of VF are distributed approximately equally among males and females, with minor fluctuations each year (64). Of the 38 hospitalized patients, there were 23 males (60.5%) and 15 females (39.5%). The greatest proportion of Arizona-wide confirmed cases and NAH inpatient cases occurred among individuals between 45 and 64 years of age. Among all 38 VF hospital-admitted patients, we observed a range of comorbidities: diabetes (16%), cancer (10%), HIV (5%), or in some cases multiple conditions (25%). Interestingly, 23% of hospitalized patients did not have notable comorbidities and were otherwise healthy (Fig. 2B). For the 38 hospitalized cases, 82% of the fungal infections were restricted to pulmonary locations, and 18% experienced disseminated infection (Fig. 2C). Nationally, dissemination occurs in between 1 and 5% of reported VF cases (18).

Next, we sought to compare racial demographics in NAH VF cases with Arizona trends. Published literature suggests that individuals with African American or Filipino backgrounds are at increased risk for severe VF (28, 66). Unfortunately, race/ethnicity is documented for only ~29% of diagnosed cases in Arizona (Table 1). This makes assessing disease risk based on race challenging and potentially inaccurate. At NAH, we were able to identify race/ethnicity in all cases (Table 1, Fig. 2C). There were 14 white (36.8%), 2 Hispanic (5.2%) and 22 American Indian/Alaskan Native (AI/AN) (57.8%) cases. The observed proportion of diagnosed cases among individuals with AI/AN heritage appears high (57.8%) despite the population prevalence (25.9%) in Coconino County and at NAH (35%) compared to other ethnic groups (Table 1). This is similar to findings released by the CDC that documented increased hospitalization in Native American populations with VF (14).

**TABLE 1:** Breakdown of unknown and known race distribution for Valley fever cases in Arizona, Coconino County, and Northern Arizona Healthcare

Total population and Valley fever cases for state, county, and hospital demographics						
Group	Arizona % (*n*)		Coconino county % (*n*)		Northern Arizona Healthcare % (*n*)	
	Population^*a*^	Valley fever^*b*^	Population^*a*^	Valley fever^*b*^	Population^*c*^	Valley fever
Unknown	n/a	72.1% (5,388)	n/a	22	1.5 % (399)	0
All groups	7,278,717	7,478	143,476	46	27,008	38
White non-Hispanic	55.38% (3,981,049)	17.6% (1,317)	63.9 % (91,649)	14	52.2 % (14,096)	36.84 % (14)
Hispanic or Latino	31.7% (2,279,253)	4.3% (325)	14.27 % (21,023)		8.3 % (2,255)	5.26 % (2)
Black or African-American	4.898% (352,121)	2.1% (159)	1.0 % (1,364)		1.2 % (324)	0
American Indian or Alaska Native	4.161% (299,123)	2.1% (157)	25.9 % (37, 187)	10	35.3 % (9,531)	57.89 % (22)
Asian or Pacific Islander	3.86% (277,474)	1.1% (84)	2.2 % (3, 149)		0.8 % (225)	0
Other/two or more	3.9% (280,574)	0.6% (48)	4.3% (6,230)		0.7% (178)	0

*^a^*ACS 2019 estimates, census.gov.

b Arizona Department of Health 2018 annual Valley fever report. Only 29% of cases’ ethnicity is known, so data are unreliable.

c Northern Arizona Healthcare, Flagstaff location, 2017, 2018.

### Northern Arizona clinical isolates within the *Coccidioides* population structure.

**FIG 3:**
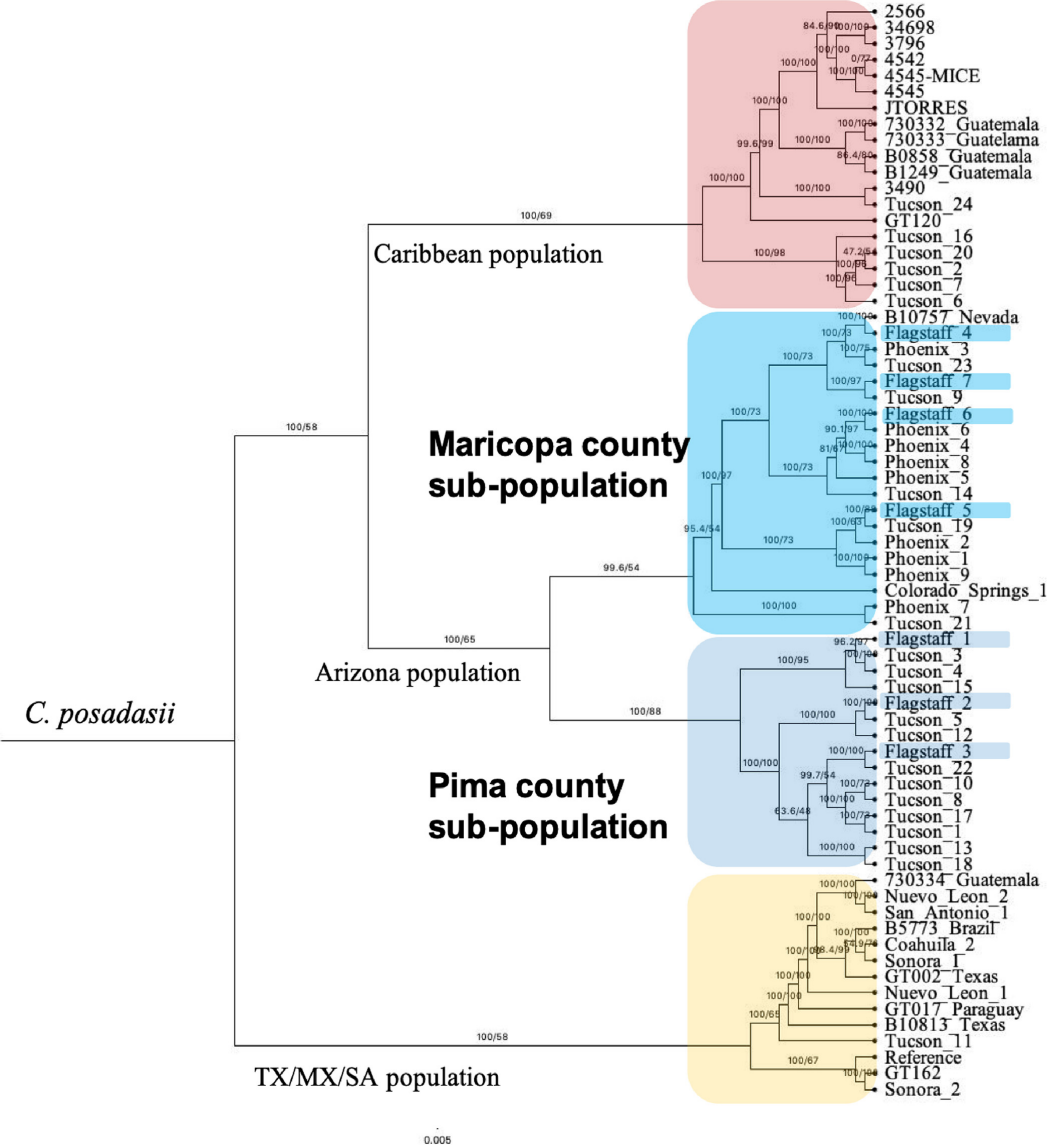
Relationship of Northern Arizona clinical isolates within the *C. posadasii* population suggests traveled related infection. The seven new clinical isolates (highlighted) group with previously published isolates recovered from patients in Maricopa or Pima county populations. *C. posadasii* comprises geographically distinct populations designated as Caribbean (top, red), Texas/Mexico and South America (bottom, yellow) and Arizona (middle, blues). Subpopulation structure in Arizona suggests genetically distinct groups within Arizona (Maricopa County; Phoenix and Pima County; Tucson). Midpoint rooted maximum likelihood tree built using 61 previously published and 7 new isolates.

We obtained seven *Coccidioides* isolates from NAH patients. We used 60 previously published whole genomes and a recently release assembly of *C. posadasii* strain Silveira reference to build an SNP (single nucleotide polymorphism)-based matrix and used the 152,663 parsimony informative SNPs to build a maximum likelihood tree (67). A *C. immitis* isolate was included in initial analysis but was separated by long branch length, and none of the seven new isolates grouped with this species (Fig. S1). Analysis with just *C. posadasii* demonstrated that the isolates obtained from patients at NAH are genetically similar to isolates from either Maricopa (*n* = 4) or Pima (*n* = 3) County, and no unique genetic cluster was found for the NAZ samples (Fig. 3).

10.1128/msphere.00352-22.7 FIG S1 Midpoint rooted maximum likelihood tree built using 62 previously published and 7 new isolates. Midpoint rooted maximum likelihood tree demonstrates the relationship of Northern Arizona clinical isolates within *C. posadasii* population and not with *C. immitis* isolate 211 from Washington. Download FIG S1, PDF file, 0.01 MB. Copyright © 2022 Mead et al. 2022 Mead et al. https://creativecommons.org/licenses/by/4.0/ This content is distributed under the terms of the Creative Commons Attribution 4.0 International license.

### *Coccidioides* spp. DNA detection in Northern Arizona soil samples.

**FIG 4:**
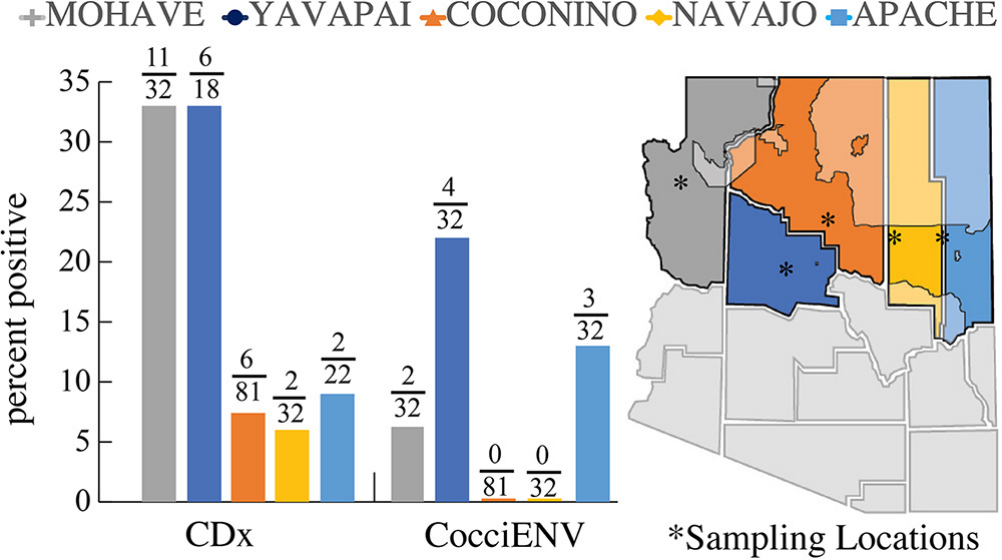
Northern Arizona soils are positive for *Coccidioides* DNA. (A) Percent positivity for soil in Mohave, Yavapai, Coconino, Navajo, and Apache counties. (B) Approximate location of soil collection sites designated with asterisk. Tribal land located in Northern Arizona counties are shaded. CDx and CocciENV are *Coccidioides*-specific PCR assays.

We surveyed 171 soil samples obtained between 2018 and 2020 from Mohave, Yavapai, Coconino, Navajo, and Apache counties for the evidence of *Coccidioides* DNA. We identified varying rates of positivity in all counties (Fig. 4, Table 2). In Mohave County there were 10/24 positive soil samples for CDx and 2/24 for both PCR assays (CDx and CocciEnv). In Yavapai County, there were 6/18 using CDx and 4/18 based on CocciENV results. In Coconino County, there were 6/75 positive CDx soils and none with CocciENV. Navajo County had 2/32 CDx positive and none with CocciENV. Lastly, Apache County had 2/22 soils that were CDx positive and 3/22 CocciENV positive. In summary, 39 samples were positive for at least one assay (Table S2), but only 6 were positive for both assays. It is common to have varying results between the two PCR methods (68, 69), and we are currently utilizing a strict filtration of both assays positive to have confidence that the soil site is likely harboring the organism. Thus, Yavapai, Apache and Mojave have stronger inference of true positive soils. In summary, our results suggest that *Coccidioides* is present in NAZ soil, although future dedicated surveys are needed to fully understand prevalence and persistence.

**TABLE 2:** Evidence for *Coccidioides* in Northern Arizona County soils

PCR results for CDX and CocciENV assays							
County	Total samples	CDx % (*n*)		CocciENV % (*n*)		CDx and CocciENV assays	
		Positive	Negative	Positive	Negative	Positive	Negative
Mohave	32	33% (11)	66% (21)	6.25% (2)	93.75% (30)	2	21
Yavapai	18	33% (6)	66% (12)	22% (4)	78% (14)	3	11
Coconino	81	7.4% (6)	92.59% (75)	0	100% (81)	0	75
Navajo	32	6% (2)	94% (30)	0% (0)	100% (30)	0	30
Apache	22	9% (2)	91% (20)	13% (3)	87% (20)	1	18
Total	171	24	147	15	147	6	155

## DISCUSSION

Environmentally acquired diseases involve complex interactions among the pathogen, the host, and the environment. Each of these factors play a role in the outcome of disease. For VF, susceptible individuals are exposed to a pathogenic fungus that originates in soil. The range of *Coccidioides* spp. appears to be expanding and is predicted to further increase in response to climate warming (59, 61, 62). In the southwestern United States, exposure is inevitable for long-term residents, and the population is growing at a faster rate than the rest of the country combined, increasing the number of naive hosts (70). Indeed, VF is on the rise in the southwestern United States (59, 61, 62, 65). To create a holistic view of VF in NAZ, we investigated documented susceptible hosts, the population structure of NAZ isolates, and the presence of the pathogen in the environment.

In Arizona, VF is a reportable disease, which allowed us to investigate current reported cases in comparison to previous trends. For each NAZ county, both total cases and cases per 100,000 population have increased (Tables S3 and 4). Although the results of our soil survey suggest the pathogen is present in the region, travel to and exposure in other highly endemic regions is possible, and probably likely (Table 2). Individuals who test positive for VF in NAZ counties share similar sex and age demographics compared to state data (Fig. 2A, Table S5). Statewide, the sex ratio for VF cases is typically 50/50 with slight variation (e.g., 48/52 in 2018) between each year (64). Individuals ages 45–64 are the most diagnosed age group with VF in Arizona, and NAZ demographics were similar. Physicians in NAZ should screen for VF, especially among individuals with respiratory symptoms that do not improve with antibacterial or antiviral treatment. In general, awareness of fungal infections including VF is low, and should be improved (9, 71, 72).

We compared 38 cases of VF treated at NAH during an 18-month period to state data to determine factors contributing to severe disease, and found similar trends related to age and sex (Table 1, Fig. 2). We identified race as a crucial, but often missing, factor during our study. In total, 57.8% (22/38) of the patients identified as AI/AN (Table 2). Sixty percent is a value higher than expected given that NAH’s typical patient population is 35% AI/AN (Table 1). We acknowledge that many socioeconomic factors can play a role in disease response, and untangling this complexity is a long-standing challenge (73–76). Importantly, evidence shows that race is a proxy for several underlying factors as it relates to disease susceptibility, and that socioeconomic status (SES), rather than race, influences health outcomes (30, 76, 77). Due to missing data, identifying an expected distribution of VF cases across racial categories is inaccurate (Table 2) (64). The racial diversity in NAZ differs from the southern parts of the state (78, 79). There are large areas of tribal land in Coconino, Navajo, and Apache counties (Fig. 1 and 2). The percentage of AI/AN individuals documented in the areas we surveyed is 26% in Coconino, 44% in Navajo, 73% in Apache, 2.2% in Mohave, and 1.6% in Yavapai counties (Table S6). In total, AI/AN make up only 4% of the Arizona population. In 1974, it was reported that tribal members had three to five times higher morbidity and mortality rates from VF compared to non-AI/AN individuals located in or near the same region (80). A follow-up paper in 1985 focused on a decreased VF dissemination in tribal members (from 8.9–3.9%); however, elevated mortality rates of native patients compared to that of white populations in the same region still remained (81). More recently, a substantial proportion of VF samples collected in New Mexico were obtained from Native American patients (63). Similarly, the Centers for Disease Control and Prevention identified that native populations suffered the highest rate of VF-associated hospitalization, compared to other races, nationwide (14). The discussion emphasized that increased dissemination and mortality rates of VF in part were due to delayed diagnosis, indicating that access to health care or other SES factors also influence disease outcomes (14, 73, 76, 82).

10.1128/msphere.00352-22.6 TABLE S6 Population by five-year age groups, county, gender, and race/ethnicity, Arizona, 2019. Download Table S6, DOCX file, 0.02 MB. Copyright © 2022 Mead et al. 2022 Mead et al. https://creativecommons.org/licenses/by/4.0/ This content is distributed under the terms of the Creative Commons Attribution 4.0 International license.

Our chart review suggests that many of the NAH patients had comorbidities such as HIV, cancer, or diabetes, which are known to increase disease susceptibility for fungal infections. However, 23% of the patients did not have any apparent immune-suppressing conditions. Among hospitalized patients, 19% had dissemination beyond the respiratory system. These dissemination rates are higher than expected (18, 33). Interestingly, 85% of these disseminated cases identified as AI/AN, supporting that these individuals may be at increased risk, as proposed previously (14, 80). Previously, health disparities for AI/AN have been documented for other diseases (82, 83). It appears that NAH is treating a higher proportion of AI/AN with VF than expected; thus, follow-up studies should be designed to appropriately assess impacts of race and SES regarding severe VF (14, 77). Our report indicates that increased risk awareness among hospitals, physicians, and local populations in NAZ is needed to increase testing for VF.

The seven isolates obtained at NAH represented a unique opportunity to investigate the local population genetics of the pathogen. Interestingly, recent work showed that isolates from New Mexico patients represent infection by both *C. immitis* and *C. posadasii* (63). Because geographic origin can be determined by genetics, we compared the NAH isolates to previously published data sets (41–43). We identified that clinical isolates from NAH are *C. posadasii* from the Arizona population. At the time of sample collection, the patients in our study resided in NAZ counties; however, travel/previous residency information was sparse or nonexistent. These findings demonstrate that these NAH patients were infected with Arizona isolate genotypes rather than a unique NAZ genetic variant. However, until isolates are obtained from the environment and whole genomes analyzed, the makeup of genetic diversity in NAZ remains unknown.

Northern Arizona’s environment is not considered preferred habitat for *Coccidioides*, based on lower case numbers, but we obtained evidence of *Coccidioides* DNA in NAZ soil. Both qPCR assays target alleles that are unique to the genus (80, 84). Six sites were positive using both assays, and we observed similar positivity rates compared to our previous soil surveys in southern Arizona (40, 53). A reasonable explanation for differential disease burden between northern and southern Arizona could be the presence of a less virulent or novel species in the region that may not cause severe disease in humans but can still be captured by the molecular assays. Reduction in virulence due to evolutionary tradeoffs related to temperature, precipitation, competition, and biodiversity are observed in other species (84, 85). Alternatively, wind-dispersed *Coccidioides* arthroconidia from southern endemic regions may have recently been established (62). The ability of arthroconidia to travel this distance and successfully propagate is unknown and difficult to model. Modeling of current and future mean annual Valley fever incidence predicts an expansion of suitable habitat, which includes NAZ (55). How increasing temperature and decreasing precipitation will impact already present novel species or wind-dispersed disease-causing strains is unknown.

In summary, our work suggests that the incidence of VF is trending upwards in NAZ. We observed that 57% of hospitalized patients were of AI/AN heritage; however. we are unable to determine if this represents an increased risk, without reliable statewide data. We identified that clinical isolates from the NAH hospital are genetically related to Southern Arizona *C. posadasii* isolates and do not originate from other populations or species found in Texas, Mexico, or California. Our soil survey data suggest that the pathogen is detectable in NAZ, which is a novel finding. Collectively, our work describes the VF disease triangle, which considers the interplay among host, pathogen, and environment in NAZ. This approach should be considered for other suspected endemic regions in which *Coccidioides* expansion is predicted. Therefore, enhanced awareness and screening for the disease is vital to the communities of NAZ and other similar regions in proximity to known endemic areas.

## MATERIALS AND METHODS

### Public health data.

Public health records were used to compare reported VF cases in NAZ counties. Yearly county case reports (2017–2019) and past averages (2012–2016) per 100,000 population were collected through the Arizona Department of Health Services (AZDHS) website (https://www.azdhs.gov/preparedness/epidemiology-disease-control/valley-fever/index.php#data-reports-publications).

### Hospital records to capture inpatient trends.

A retrospective chart review identified all in-patients who were screened for VF during an 18-month period (1 July 2017 to 31 December 2018) at the Flagstaff location of Northern Arizona Healthcare (NAH). A potential case was suspected when a patient was subjected to certain evaluations through X-ray, serological titer, or complement fixation serological diagnostics. Confirmed positive diagnoses were defined as a positive IgM and/or CF titer >1:8. All clinical tests were performed by NAH clinical lab following IDSA guidelines and were reviewed by the infectious disease department to confirm.

### Genomic DNA from clinical isolates from NAH.

The fungal clinical isolates were previously collected and stored under Institutional Review Board No. 764034 as part of the Northern Arizona University Biobank with NAH. The isolates (Table S1) were transferred to NAU’s biosafety level 3 laboratory, and handled as previously described (86, 87).

10.1128/msphere.00352-22.1 TABLE S1 Metadata for Flagstaff isolates. Download Table S1, XLSX file, 0.05 MB. Copyright © 2022 Mead et al. 2022 Mead et al. https://creativecommons.org/licenses/by/4.0/ This content is distributed under the terms of the Creative Commons Attribution 4.0 International license.

Fungal isolates were cultured as previously described (87, 88). Briefly, pure cultures were grown on 2xGYE (2% glucose, 1% yeast extract, 2% agar wt/vol) at 30°C, then used to inoculate liquid 2xGYE (2% glucose, 1% yeast extract) in vented baffled Erlenmeyer flasks and grown at 30°C shaking for 6 days. These cultures were heat inactivated at 80°C for 30 min in fungal lysis buffer. Genomic DNA was obtain using traditional phenol:choloroform solvents and precipitated using isopropanol and EtOH. DNA was visualized on a 1% agarose gel. Sequencing occurred on a MiSeq V3 600 cycle kit. Raw reads have been deposited in the NCBI SRA database under accession number PRJNA722304.

Raw reads for 60 previously published *C. posadasii* samples representing the Caribbean, Arizona, Texas/Mexico/South America clades (41), and the seven new clinical isolates were aligned (82.72% or 23,336,819 bases) to reference the *C. posadasii* strain Silveira genome, accession number PRJNA494320 (89). The Northern Arizona Sequencing Pipeline (90) was used to build an SNP (single nucleotide polymorphism) matrix and map 270,853 SNPs, which were used to identify the best substitution model TVM+F+ASC+R5 using 1,000 bootstrap iterations and build a maximum likelihood tree using IQ-TREE version 1.6.1, based on 152,663 parsimony-informative SNPs (91). The phylogenetic tree distribution was visualized using Figtree v1.4.4 (92). A similar approach using *C. immitis* strain WA-211 (93) as an outgroup was used to ensure no *C. immitis* was identified among the new isolates (Fig. S1).

### Environmental detection of coccidioides.

Soil samples (*n* = 171) were collected between 2018 and 2020 (Table S2). We target animal burrows and areas with rodent activity based on Kollath et al., 2020 (53). Soils were collected and processed as previously described (53). Briefly, each sample was collected with a disinfected garden trowel and stored in sterile 50-mL collection containers at room temperature until processing. DNA was extracted using the Qiagen DNeasy Powersoil Pro Kit (Qiagen, Boston, MA, USA) following manufacturer’s protocol and adding a 65C heating step during lysis. The presence or absence of *Coccidioides* spp. DNA in soil samples was determined by two highly specific TaqMan-based CocciDxQ and CocciENV real-time qPCR assays (68, 69) using a Quant Studio 12k Flex real-time PCR system (ThermoFisher Scientific). Three technical real-time PCR replicates were run for each soil DNA sample. A reaction was considered positive if it showed logarithmic amplification and produced a C_T_ value of <40 for all three replicates.

10.1128/msphere.00352-22.2 TABLE S2 Description of collection dates, location, and PCR results for soil samples. Download Table S2, XLSX file, 0.02 MB. Copyright © 2022 Mead et al. 2022 Mead et al. https://creativecommons.org/licenses/by/4.0/ This content is distributed under the terms of the Creative Commons Attribution 4.0 International license.

### Study limitations.

To our knowledge, this study is the first attempt to consider the interaction of host, pathogen, and environment that contribute to VF in NAZ. Therefore, results and discussion presented here are an estimate, with need for further data collection. Our goal was to document VF trends in NAZ with data at hand. We present VF cases and cases per 100,000 to account for reported cases and population fluctuation. However, we acknowledge that the number of tests administered can impact these numbers, but we did not have access to these data. County data and inpatient hospital data do not reflect asymptomatic cases, individuals without access to medical care, or misdiagnosis. Ideally, the retrospective chart review would encompass a longer time span; however, due to the COVID-19 pandemic, we chose to present the current data rather than request further hospital resources. Lastly, the PCR methods utilized in this study detect *Coccidioides* DNA, which cannot confirm the presence of live infectious organisms nor provide phylogenetic placement of NAZ soil strains.
