# Supplementary material for: Coccidioidomycosis in Northern Arizona: an Investigation of the Host, Pathogen, and Environment Using a Disease Triangle Approach
Source: mSphere. 2022 Aug 16;7(5):e00352-22. doi: 10.1128/msphere.00352-22 (PMC9599602; doi:10.1128/msphere.00352-22)
Supplement: FIG S1 [file msphere.00352-22-s0007.pdf]

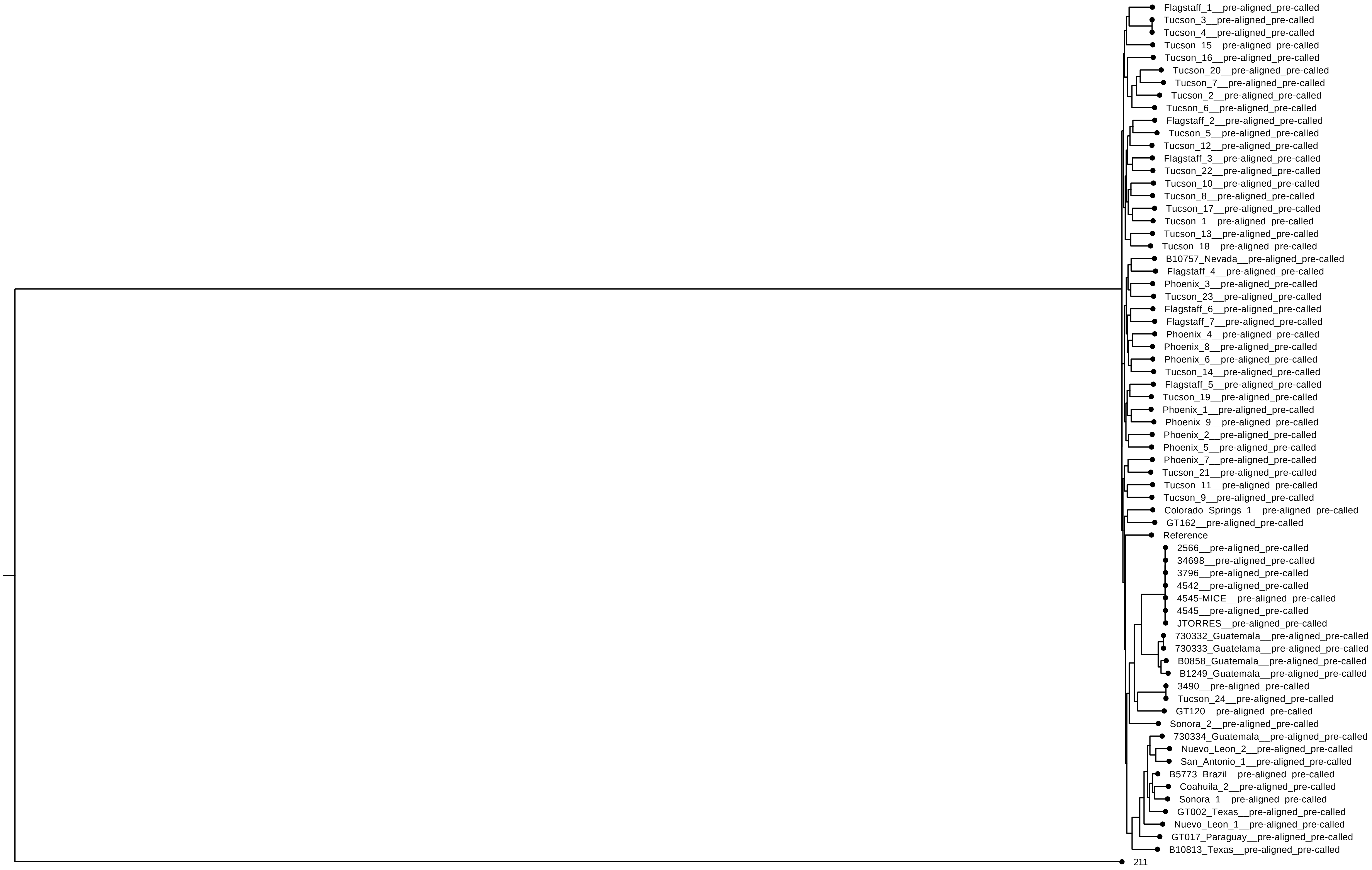
Flagstaff_1__pre-aligned_pre-called
Tucson_3__pre-aligned_pre-called
Tucson_4__pre-aligned_pre-called
Tucson_15__pre-aligned_pre-called
Tucson_16__pre-aligned_pre-called
Tucson_20__pre-aligned_pre-called
Tucson_7__pre-aligned_pre-called
Tucson_2__pre-aligned_pre-called
Tucson_6__pre-aligned_pre-called
Flagstaff_2__pre-aligned_pre-called
Tucson_5__pre-aligned_pre-called
Tucson_12__pre-aligned_pre-called
Flagstaff_3__pre-aligned_pre-called
Tucson_22__pre-aligned_pre-called
Tucson_10__pre-aligned_pre-called
Tucson_8__pre-aligned_pre-called
Tucson_17__pre-aligned_pre-called
Tucson_1__pre-aligned_pre-called
Tucson_13__pre-aligned_pre-called
Tucson_18__pre-aligned_pre-called
B10757_Nevada__pre-aligned_pre-called
Flagstaff_4__pre-aligned_pre-called
Phoenix_3__pre-aligned_pre-called
Tucson_23__pre-aligned_pre-called
Flagstaff_6__pre-aligned_pre-called
Flagstaff_7__pre-aligned_pre-called
Phoenix_4__pre-aligned_pre-called
Phoenix_8__pre-aligned_pre-called
Phoenix_6__pre-aligned_pre-called
Tucson_14__pre-aligned_pre-called
Flagstaff_5__pre-aligned_pre-called
Tucson_19__pre-aligned_pre-called
Phoenix_1__pre-aligned_pre-called
Phoenix_9__pre-aligned_pre-called
Phoenix_2__pre-aligned_pre-called
Phoenix_5__pre-aligned_pre-called
Phoenix_7__pre-aligned_pre-called
Tucson_21__pre-aligned_pre-called
Tucson_11__pre-aligned_pre-called
Tucson_9__pre-aligned_pre-called
Colorado_Springs_1__pre-aligned_pre-called
GT162__pre-aligned_pre-called
Reference
2566__pre-aligned_pre-called
34698__pre-aligned_pre-called
3796__pre-aligned_pre-called
4542__pre-aligned_pre-called
4545-MICE__pre-aligned_pre-called
4545__pre-aligned_pre-called
JTORRES__pre-aligned_pre-called
730332_Guatemala__pre-aligned_pre-called
730333_Guatelama__pre-aligned_pre-called
B0858_Guatemala__pre-aligned_pre-called
B1249_Guatemala__pre-aligned_pre-called
3490__pre-aligned_pre-called
Tucson_24__pre-aligned_pre-called
GT120__pre-aligned_pre-called
Sonora_2__pre-aligned_pre-called
730334_Guatemala__pre-aligned_pre-called
Nuevo_Leon_2__pre-aligned_pre-called
San_Antonio_1__pre-aligned_pre-called
B5773_Brazil__pre-aligned_pre-called
Coahuila_2__pre-aligned_pre-called
Sonora_1__pre-aligned_pre-called
GT002_Texas__pre-aligned_pre-called
Nuevo_Leon_1__pre-aligned_pre-called
GT017_Paraguay__pre-aligned_pre-called
B10813_Texas__pre-aligned_pre-called
211
