# Supplementary material for: Coccidioidomycosis in Northern Arizona: an Investigation of the Host, Pathogen, and Environment Using a Disease Triangle Approach
Source: mSphere. 2022 Aug 16;7(5):e00352-22. doi: 10.1128/msphere.00352-22 (PMC9599602; doi:10.1128/msphere.00352-22)
Supplement: TABLE S6 [file msphere.00352-22-s0006.docx]

Supplemental Table 6

| POPULATION BY FIVE-YEAR AGE GROUPS, COUNTY, GENDER, AND RACE/ETHNICITY, ARIZONA, 2019 | | | | | | | |
| --- | --- | --- | --- | --- | --- | --- | --- |
|  | | | | **Total** | | | **Percent** |
| **Arizona** | **All groups** | | **Total** | 7,189,020 | | | 100.0 |
|  |  |  | Male | 3,574,659 | | | 49.7 |
|  |  |  | Female | 3,614,361 | | | 50.3 |
|  | White non-Hispanic | | Total | 3,981,049 | | | 55.4 |
|  |  |  | Male | 1,968,918 | | | 27.4 |
|  |  |  | Female | 2,012,131 | | | 30.0 |
|  | Hispanic or Latino | | Total | 2,279,253 | | | 31.7 |
|  |  |  | Male | 1,146,980 | | | 16.0 |
|  |  |  | Female | 1,132,273 | | | 15.8 |
|  | Black or African American | | Total | 352,121 | | | 4.9 |
|  |  |  | Male | 182,011 | | | 2.6 |
|  |  |  | Female | 170,110 | | | 2.4 |
|  | American Indian or Alaska Native | | Total | 299,123 | | | 4.2 |
|  |  |  | Male | 145,181 | | | 2.0 |
|  |  |  | Female | 153,942 | | | 2.1 |
|  | Asian or Pacific Islander | | Total | 277,474 | | | 3.7 |
|  |  |  | Male | 131,569 | | | 1.8 |
|  |  |  | Female | 145,905 | | | 2.0 |
| POPULATION BY FIVE-YEAR AGE GROUPS, COUNTY, GENDER, AND RACE/ETHNICITY, ARIZONA, 2019 | | | | | | | |
| **Apache** | **Total** | | **Total** | | **Total** | **Percent** | |
|  |  |  |  |  | 71,808 | 0.99 | |
|  |  |  | Male | | 35,307 | 49.2 | |
|  |  |  | Female | | 36,501 | 50.8 | |
|  | White non-Hispanic | | Total | | 13,313 | 18.5 | |
|  | Hispanic or Latino | | Total | | 4,589 | 6.39 | |
|  | Black or African American | | Total | | 640 | 0.9 | |
|  | American Indian or Alaska Native | | Total | | 52,923 | **73.7** | |
|  | Asian or Pacific Islander | | Total | | 343 | 0.48 | |
| **Coconino** | **Total** | | **Total** | | **Total** | **Percent** | |
|  |  |  |  |  | 147,275 | 2.0 | |
|  |  |  | Male | | 72,778 | 49.4 | |
|  |  |  | Female | | 74,497 | 50.6 | |
|  | White non-Hispanic | | Total | | 81,209 | 55.1 | |
|  | Hispanic or Latino | | Total | | 21,023 | 14.3 | |
|  | Black or African American | | Total | | 2,496 | 1.7 | |
|  | American Indian or Alaska Native | | Total | | 39,187 | 26.6 | |
|  | Asian or Pacific Islander | | Total | | 3,360 | 2.3 | |
| **Navajo** | **Total** |  | | | **Total** | **Percent** | |
|  |  | Total | | | 112,825 | 1.6 | |
|  |  | Male | | | 56,472 | 50.1 | |
|  |  | Female | | | 56,353 | 49.9 | |
|  | White non-Hispanic | Total | | | 47,698 | 42.3 | |
|  | Hispanic or Latino | Total | | | 12,928 | 11.5 | |
|  | Black or African American | Total | | | 1,348 | 1.2 | |
|  | American Indian or Alaska Native | Total | | | 49,995 | 44.3 | |
|  | Asian or Pacific Islander | Total | | | 856 | 0.76 | |
| **Mohave** | **RACE/ETHNICITY** | | **Total** | | **Total** | **Percent** | |
|  |  |  |  |  | 216,985 |  | |
|  |  |  | Male | | 109,728 | 50.6 | |
|  |  |  | Female | | 107,257 | 49.4 | |
|  | White non-  Hispanic | | Total | | 169,143 | 77.9 | |
|  | Hispanic or  Latino | | Total | | 36,533 | 16.8 | |
|  | Black or  African  American | | Total | | 3,028 | 1.4 | |
|  | American  Indian or  Alaska Native | | Total | | 4,941 | 2.3 | |
|  | Asian or Pacific Islander | | Total | | 3,340 | 1.5 | |
| **Yavapai** | **RACE/ETHNICITY** | | **Total** | | **Total** | **Percent** | |
|  |  |  |  |  | 232,386 | 3.2 | |
|  |  |  | Male | | 113,515 | 48.8 | |
|  |  |  | Female | | 118,871 | 51.2 | |
|  | White non-  Hispanic | | Total | | 189,145 | 81.4 | |
|  | Hispanic or  Latino | | Total | | 34,115 | 14.7 | |
|  |  |  | Male | | 17,529 | 7.5 | |
|  |  |  | Female | | 16,586 | 7.1 | |
|  | Black or  African American | | Total | | 2,279 | 0.98 | |
|  | American  Indian or  Alaska Native | | Total | | 3,800 | 1.6 | |
|  | Asian or  Pacific Islander | | Total | | 3,047 | 1.3 | |
